# Giant Piezoresistive Effect and Strong Bandgap Tunability in Ultrathin InSe upon Biaxial Strain

**DOI:** 10.1002/advs.202001645

**Published:** 2020-08-20

**Authors:** Qinghua Zhao, Tao Wang, Riccardo Frisenda, Andres Castellanos‐Gomez

**Affiliations:** ^1^ State Key Laboratory of Solidification Processing Northwestern Polytechnical University Xi'an 710072 P. R. China; ^2^ Key Laboratory of Radiation Detection Materials and Devices Ministry of Industry and Information Technology Xi'an 710072 P. R. China; ^3^ Materials Science Factory Instituto de Ciencia de Materiales de Madrid (ICMM‐CSIC) Madrid E‐28049 Spain

**Keywords:** bandgap tunability, biaxial strains, InSe, photoluminescence, piezoresistive effects, Raman spectroscopy

## Abstract

The ultrathin nature and dangling bonds free surface of 2D semiconductors allow for significant modifications of their bandgap through strain engineering. Here, thin InSe photodetector devices are biaxially stretched, finding, a strong bandgap tunability upon strain. The applied biaxial strain is controlled through the substrate expansion upon temperature increase and the effective strain transfer from the substrate to the thin InSe is confirmed by Raman spectroscopy. The bandgap change upon biaxial strain is determined through photoluminescence measurements, finding a gauge factor of up to ≈200 meV %^−1^. The effect of biaxial strain on the electrical properties of the InSe devices is further characterized. In the dark state, a large increase of the current is observed upon applied strain which gives a piezoresistive gauge factor value of ≈450–1000, ≈5–12 times larger than that of other 2D materials and of state‐of‐the‐art silicon strain gauges. Moreover, the biaxial strain tuning of the InSe bandgap also translates in a strain‐induced redshift of the spectral response of the InSe photodetectors with Δ*E*_cut‐off_ ≈173 meV at a rate of ≈360 meV %^−1^ of strain, indicating a strong strain tunability of the spectral bandwidth of the photodetectors.

## Graphical abstract

2D InSe is particularly suited for strain engineering. In this work, thin InSe photodetector devices are biaxially stretched, finding a giant change of their resistance upon strain and a bandgap tunability, determined through photoluminescence measurements, up to ≈200 meV %^−1^ of biaxial strain.

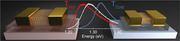

Strain engineering, the modification of the optical, magnetic, electrical, and optoelectronic properties of a given material by applying an external mechanical deformation to its crystal lattice, is establishing itself as one of the most prospective strategies to controllably modify the properties of 2D materials.^[^ ^1^ ^]^ In fact, the lack of dangling bonds on their surface makes them extremely resilient to the mechanical deformation without fracture,^[^ ^2^ ^]^ even approaching the theoretical limit (predicted by Griffith) for defect‐free materials.^[^ ^3^ ^]^ The capability of applying very large deformations together with strain sensitive band‐structures makes of 2D materials a very suitable family of materials for strain engineering. Based on this outstanding stretchability and strain engineered band‐structure, novel strain‐tunable devices for information, sensor, and energy‐saving technologies, usually referred as straintronics,^[^ ^4^ ^]^ have been recently reported. In fact, very recently flexible broadband photodetectors based on continuous strain modulation,^[^ ^5^ ^]^ micro stress sensors,^[^ ^6^ ^]^ atomic‐thin nanogenerators based on piezotronics,^[^ ^7^ ^]^ and spatially and spectrally isolated quantum emitters on a prepatterned rigid substrate have been achieved.^[^ ^8^ ^]^

During the last years, multiple works studying the strain tunability of the bandgap of several 2D semiconductors, including transition metal dichalcogenides (TMDCs), black phosphorus (bP), and other 2D semiconductors, have been reported (**Table** **1**).^[^ ^5^, ^6^, ^9^ ^]^ Very recently, InSe has shown sizeable larger strain tunability with respect to TMDCs and black phosphorus upon uniaxial strain loading and local strain modification.^[^ ^9^, ^10^ ^]^ According to the works reported for TMDCs and bP, biaxial strain usually yields stronger bandgap tunability than uniaxial strain because of the larger lattice deformation in both crystal orientations.^[^ ^9ae^ ^]^ Although recent calculations also predicted that biaxial strain should have a stronger effect on the InSe band structure than uniaxial strain,^[^ ^11^ ^]^ its experimental realization is still lacking.

**Table 1:** Summarized comparison of bandgap tunability of 2D semiconductors under strain engineering. The *ε* ^max^ refers to the maximum strain applied on the flexible substrate or directly on a suspended 2D flake or induced by a prepatterned substrate. ^(D)^ and ^(I)^ indicate direct and indirect bandgap, respectively

Materials	Strain type	Method/substrate	Δ*E*_g_^max^, ɛ^max^	Gauge factor [meV %^−1^]	Ref.
1L MoS_2_	Uniaxial	Mechanical bending, PC	−81 meV, 1.8%	−45 ± 7	^[^ ^9a^ ^]^
		Mechanical bending, PMMA	−33 meV, 0.52%	−64 ± 5	^[^ ^9b^ ^]^
		Mechanical bending, PET	−38.4 meV, 0.8%	−48	^[^ ^9c^ ^]^
		Mechanical bending, PC	−57.5 meV, 1.37%	−42	^[^ ^9d^ ^]^
		Mechanical bending, PC	−44.5 meV, 1.06%	−42	^[^ ^9e^ ^]^
		MEMS mechanics, suspended	−49.4 meV, 1.3%	−38 ± 1^a)^	^[^ ^9f^ ^]^
		Mechanical bending, PVA	−300 meV, 1.7%	−125^b)^, −61	^[^ ^9g^ ^]^
		Mechanical bending, PET	−36 meV, 0.64%	−56	^[^ ^9h^ ^]^
		Substrate stretching, PDMS	−15 meV, 4.8%	−3^a)^	^[^ ^9i^ ^]^
		Mechanical bending, PI	−31 meV, 0.4%	−78 ± 4	^[^ ^9j^ ^]^
	Biaxial	Thermal expansion, PC	−65 meV, 0.48%	−135	^[^ ^5a^ ^]^
		Pressurized membranes, suspended	−500 meV, 5%	−99	^[^ ^9k^ ^]^
		Thermal expansion, PDMS, PP	−12.5 meV, −51.1 meV, 1%	−12.5, −51.1	^[^ ^9l^ ^]^
		Prepatterned substrate, SiO_2_	−50 meV, 0.565%	−110	^[^ ^9n^ ^]^
		AFM indentation, suspended	–, 7%	−77.3 ± 10	^[^ ^6c^ ^]^
2L MoS_2_	Uniaxial	Mechanical bending, PC	−32 meV^(D)^, −77 meV^(I)^, 0.6%	−53 ± 10^(D)^, −129 ± 20^(I)^	^[^ ^9a^ ^]^
		Mechanical bending, PMMA	−25 meV, 0.52%	−48 ± 5	^[^ ^9b^ ^]^
		Mechanical bending, PET	−36.8 meV^(D)^, −68.8 meV^(I)^, 0.8%	−46^(D)^, −86^(I)^	^[^ ^9c^ ^]^
		Mechanical bending, PC	−78 meV, 1.6%	−49 ± 1	^[^ ^9ad^ ^]^
		Mechanical bending, PI	−12 meV^(D)^, 56 meV^(I)^, 0.36%	−34 ± 3^(D)^, −155 ± 11^(I)^	^[^ ^9j^ ^]^
	Biaxial	AFM indentation, suspended	–, 7%	−116.7 ± 10	^[^ ^6c^ ^]^
3L MoS_2_	Uniaxial	Prestrained substrate, Gel‐Film	−90 meV, 2.5%	−36	^[^ ^9o^ ^]^
	Biaxial	piezoelectric substrate, PMN‐PT	−60 meV, 0.2%	−300	^[^ ^9m^ ^]^
		AFM indentation, suspended	–, 7%	−22.7 ± 6	^[^ ^6c^ ^]^
1L MoSe_2_	Uniaxial	Mechanical bending, PC	−40.7 meV, 1.07%	−38 ± 2	^[^ ^9d^ ^]^
		Mechanical bending, PC	−30 meV, 1.1%	−27 ± 2	^[^ ^9p^ ^]^
		Mechanical bending, PEN	−28 meV, 0.5%	−54.8 ± 5.8	^[^ ^9q^ ^]^
	Biaxial	Thermal expansion, PP	−33 meV, 1%	−33	^[^ ^9l^ ^]^
1L WS_2_	Uniaxial	Mechanical bending, PC	−69 meV, 1.26%	−55 ± 2	^[^ ^9d^ ^]^
		Mechanical bending, PVA	−253 meV, 5.68%	−43^a)^	^[^ ^9g^ ^]^
		Mechanical bending, PET	−27.5 meV, 0.64%	−43	^[^ ^9h^ ^]^
		Mechanical bending, PEN	−31 meV, 0.5%	−61.2 ± 3.8	^[^ ^9q^ ^]^
		Mechanical bending, PET	−44 meV^(D)^, −76 meV^(I)^, 4%	−11^a)^ ^(D)^, −19^a)^ ^(I)^	^[^ ^9r^ ^]^
		Substrate stretching, PDMS	−20 meV, 16%	−1.3^a)^	^[^ ^9s^ ^]^
	Biaxial	Thermal expansion, PP	−95 meV, 1%	−95	^[^ ^9l^ ^]^
1L WSe_2_	Uniaxial	Mechanical bending, PC	−72.5 meV, 1.48%	−49 ± 2	^[^ ^9d^ ^]^
		Mechanical bending, PVA	−176 meV, 1.7% −137 meV, 2.56%	−109 −53^a)^	^[^ ^9g^ ^]^
		Mechanical bending, PEN	−20 meV, 0.35%	−53 ± 3.1	^[^ ^9q^ ^]^
		Mechanical bending, PC	−75.5 meV, 1.4%	−54	^[^ ^9t^ ^]^
		Mechanical bending, PETG	101 meV, 2.1%	−48	^[^ ^9u^ ^]^
	Biaxial	Thermal expansion, PP	−63 meV, 1%	−63	^[^ ^9l^ ^]^
2L WSe_2_	Uniaxial	Mechanical bending, PETG	−68 meV, 1.51%	−45	^[^ ^9v^ ^]^
		Mechanical bending, PET	−45 meV^(D)^, −40 meV^(I)^, 2%	−22.5^(D)^, 20^(I)^	^[^ ^9w^ ^]^
		Mechanical bending, PETG	−110 meV, 2.1%	−52	^[^ ^9x^ ^]^
1L ReSe_2_	Uniaxial	Prestrained substrate, Gel‐Film	−70 meV, 1.64%	−43	^[^ ^9y^ ^]^
6L bP	Uniaxial	Mechanical bending, PET	110 meV, 0.92%	120	^[^ ^9z^ ^]^
18L bP	Uniaxial	Prestrained substrate, Gel‐Film	700 meV, 5%	100–140	^[^ ^9aa^ ^]^
6L bP	Uniaxial	Mechanical bending, PP	132 meV, 1%	132	^[^ ^9ae^ ^]^
	Biaxial	Thermal expansion, PP	67 meV, 0.3%	222	
4–8L InSe	Uniaxial	Mechanical bending, PP	−110 meV, 1.15%	−(90–100)	^[^ ^9ab^ ^]^
4–35 nm InSe	Uniaxial	Mechanical bending, PET	−118 meV, 1.06%	−(80–150)	^[^ ^9ac^ ^]^
5L InSe	Biaxial	Thermal expansion, PC	−26 meV, 0.13%	−200	^This work^

a) Data obtained based on CVD grown 2D materials.

b) Data obtained in a polyvynilacetate (PVA) encapsulated sample.

Here we experimentally study the bandgap modification in ultrathin InSe by biaxial strain. We fabricate InSe photodetectors onto polycarbonate (PC) allowing us to control the applied biaxial strain through the substrate expansion upon temperature increase. Through Raman spectroscopy we verify that biaxial strain is effectively transduced from the substrate expansion. Photoluminescence (PL) measurements are used to probe the effect of biaxial strain tuning of the InSe bandgap. We found a strong thickness dependence of the strain‐tunability of the bandgap, reaching ≈200 meV %^−1^ of biaxial strain for ultrathin (≈5 layers) InSe flakes. With electrical transport measurements we found a large increase of the dark current upon biaxial straining giving a piezoresistive gauge factor of GF ≈ 450 to 1000, that can reach ≈5–12 times larger than that of other 2D materials and of state‐of‐the‐art silicon strain gauges (GF ≈ 200).^[^ ^6^, ^12^ ^]^ Interestingly, biaxial strain also has a strong effect on the spectral response of our photodetector, redshifting the photocurrent spectra up to ≈173 meV at a rate of ≈360 meV %^−1^ of strain, indicating a very strong strain tunable spectral bandwidth.

The Au–InSe–Au devices are fabricated by mechanical exfoliation of bulk InSe single crystals grown by the Bridgman method (the characterizations of bulk crystals have been reported in our previous work) with Nitto SPV 244 tape.^[^ ^13^ ^]^ The cleaved crystallites are then transferred onto a Gel‐Film (Gel‐Pak, WF 6.0 mil × 4) stamp. Quantitative optical microscopy is used to identify and select ultrathin InSe flakes on the Gel‐Film stamp. Then the selected flake is deterministically placed bridging a pair of gold electrodes prepatterned on a target PC substrate.^[^ ^14^ ^]^ Subsequently, a larger thin h‐BN flake (30–50 layers) is placed on the top of the active region in the device to provide a full insulating encapsulation to slow down the environmental induced degradation of InSe.^[^ ^15^ ^]^ Note that all these fabrication steps are carried out under ambient conditions within 30 min. **Figure** **1** shows the details of the fabrication of an Au–InSe–Au device on the PC substrate. Figure 1a shows the schematic (top panel) and optical images obtained with reflection (middle panel) and transmission (bottom panel) mode of a selected ultrathin ≈ 20 nm InSe flake (shown by inset picture) deterministically transferred bridging two 50 nm Au/ 5 nm Ti electrodes prepatterned on the surface of PC substrate. Figure 1b shows the geometry (top panel) and pictures (middle and bottom panels) of the final devices after top encapsulation with h‐BN. We chose a PC substrate because of the combination of its high thermal expansion (to yield sizeable biaxial strain upon heating, *α* = 64 × 10^−6^ °C^−1^) and its high Young's modulus (to ensure a good strain transfer, *E* = 2.5 GPa).^[^ ^5^, ^9^ ^]^ We also fabricated a set of InSe devices on SiO_2_/Si substrates (see Figure S1 in the Supporting Information) that have negligible thermal expansion coefficient (*α* < 1×10^−6 ^°C^−1^).^[^ ^16^ ^]^ This set of devices is used as control samples to determine the role of the intrinsic temperature increase, without biaxial strain, on the observed features. This allows for the disentanglement of the temperature and strain effects on the observed features during the measurements. To passivate the defects existing in thin InSe flakes, thanks to the air species trapped at the interfaces, and reach a long‐term stable working state with fast photodetection operation (as shown in Figures S2 and S3 in the Supporting Information),^[^ ^17^ ^]^ all the Au–InSe–Au devices have been annealed in situ in air at ≈100 °C for around 2 h on the microheater mounted on probe station before carrying out the Raman spectroscopy and optoelectronic characterizations discussed in this work.^[^ ^18^ ^]^ In Figures S4 and S5 in the Supporting Information, we show how the current flowing through the devices evolves under 530 nm global illumination during the annealing process both on PC and SiO_2_/Si substrates, as expected for a defects passivation process in InSe photodetector. Raman spectroscopy measurement in Figure S6 in the Supporting Information indicates there is no structural change before and after annealing.

**Figure 1:**
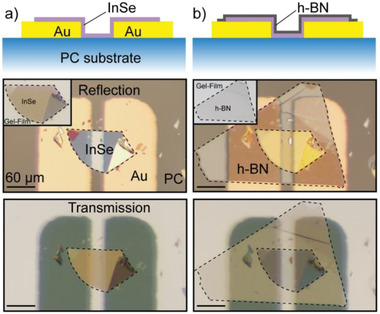
Fabrication of Au–InSe–Au device on polycarbonate (PC) substrate. Schematics (top) and optical microscopy images of an Au–InSe–Au device a) before and b) after encapsulating a h‐BN flake on the surface with optical microscope reflection (middle) and transmission (bottom) mode. Insets: the optical microscopy images of selected thin InSe flake and h‐BN fabricated on Gel‐Film observed with transmission mode.

We first employ Raman spectroscopy to characterize the strain transfer from the PC substrate to the flake upon thermal expansion. **Figure** **2**a shows Raman spectra acquired in the 20 nm thick InSe device at different PC substrate temperatures (from ≈26 to ≈100 °C), corresponding to a biaxial thermal expansion ranging from 0% up to 0.48%.^[^ ^5^, ^9^ ^]^ We address the reader to Figure S7 in the Supporting Information for a second set of Raman measurements acquired on the same sample during another heating cycle to demonstrate the reproducibility of the thermal induced biaxial straining approach. Three Raman active in‐plane modes *A*_1_′(1), *A*_2_′′(1), and *A*_2_′(1) located at ≈113, ≈198, and ≈226 cm^−1^, and one out‐of‐plane *E*′(2) located at ≈176 cm^−1^ are observed, which is consistent with hexagonal crystal structure of ultrathin InSe with *ε* stacking sequence.^[^ ^13^, ^17^, ^18^, ^19^ ^]^ All the Raman peaks shift toward lower Raman shifts upon thermal expansion, similar to recently reported experimental works on uniaxial strained InSe due to phonon softening.^[^ ^9^, ^10^, ^20^ ^]^ That is the increase of the covalent bonds length introduced by the applied tensile strain results in a weaker restoring force of vibrations, and thus lower phonon frequencies. As a control experiment we repeat the same measurements on an InSe device fabricated on SiO_2_/Si (with negligible thermal expansion). In this control sample the Raman peaks position shift at much lower rate upon SiO_2_/Si substrate temperature increase (see Figure 2b), indicating that the shift observed in the PC based device can be mostly attributed to the effect of biaxial strain. By subtracting the shift obtained on the SiO_2_/Si substrate to that of the PC substrate, in Figure 2c we can determine the redshift rate due to biaxial strain: −1.48, −4.84, −5.32, and −5.77 cm^−1^ %^−1^ of biaxial strain for the *A*_1_′ (1), *E*′′ (2), *A*_2_′′ (1), and *A*_2_′ (1) Raman modes, respectively. We address the reader to the Figure S8 in the Supporting Information for another dataset acquired on a 13 nm thick InSe flake showing very similar Raman peak shift upon straining, demonstrating that a similar strain transfer is achieved for the 20 and the 13 nm InSe flakes. We attribute this good strain transfer, even for relatively thick InSe flakes, to the low Young's modulus of InSe (*E* = 23 ± 5 GPa,^[^ ^13^ ^]^ 10–20 times smaller than that of transition metal dichalcogenides, TMDCs)^[^ ^21^ ^]^ as strain transfer from the substrate to the flake is inversely proportional to the Young's modulus of the flake. Indeed, finite element calculations predict a strain transfer of ≈100% for InSe on PC substrates.^[^ ^13^ ^]^ In order to probe if the top h‐BN encapsulation has any effect on the strain transfer we have performed a control straining experiment on a ≈13 layers InSe flake that has been partially encapsulated with h‐BN (see Figure S9 in the Supporting Information), finding very similar results on the unencapsulated and on the encapsulated parts.

**Figure 2:**
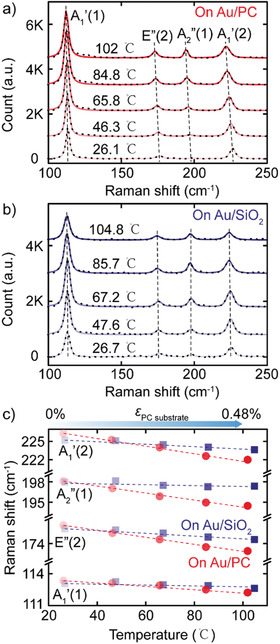
Temperature‐dependent Raman Spectroscopy of thin InSe flakes deposited on Au/PC and on Au/SiO_2_/Si substrates. Raman spectra of thin InSe recorded on a) Au/PC and b) on Au/280 nm SiO_2_/Si substrates with 50× objective as a function of temperature (from ≈26 to ≈ 100 °C). c) Temperature‐dependency of four Raman active modes (*A*_1_′ (1), *E*′′ (2), *A*_2_′′ (1) and *A*_1_′ (1)) of thin InSe on PC (red) and on Au/280 nm SiO_2_/Si (blue) substrate. The top axis in (c) indicates the biaxial strain induced by the thermal expansion of the PC substrate.

The redshift rates of the Raman peaks are around two times larger than the value reported for uniaxial strained thin InSe.^[^ ^9^, ^10^ ^]^ This information can be highly valuable as Raman spectroscopy is commonly used to monitor residual or built‐in strains during the device fabrication and/or growth of other 2D materials. More interestingly, we can further calculate the Grüneisen parameters,^[^ ^22^ ^]^ that describes the effect of a volume change on the vibrational properties, of the *A*_1_′ (1), *E*′′ (2), *A*_2_′′ (1), and *A*_2_′ (1) Raman modes by using the obtained Raman mode shift rate, which take the values of 0.65, 1.38, 1.34, and 1.28. These values are comparable with those reported in the literature for uniaxial strained InSe.^[^ ^9ac^ ^]^ The determination of the Grüneisen parameters through biaxial straining, however, has the advantage that (unlike in uniaxial strain) no assumptions about the Poisson's ratio value are needed.

We study the effect of the applied biaxial strain on the bandgap of InSe through PL. **Figure** **3**a shows PL spectra acquired on InSe flakes transferred onto a PC substrate and onto a SiO_2_/Si substrate at different temperatures. The PL spectra show a peak corresponding to the direct bandgap transition at the Γ point of the Brillouin zone and it is thus a good probe of the bandgap of InSe.^[^ ^9^, ^23^ ^]^ Note that one can use the PL energy emission to determine the number of layers of InSe (see Figure S10 in the Supporting Information). As for the Raman experiments, we use the measurements on the SiO_2_/Si substrates as a control experiment to probe the intrinsic shift of the PL peaks upon temperature increase (without biaxial strain). This allows to determine the biaxial strain induced PL shift, subtracting the PL shift measured on SiO_2_/Si substrates (only thermal contribution) to the PL shift measured on PC substrates (thermal + biaxial strain contribution). Figure 3a shows how the PL shift on PC substrates is much larger than that measured on SiO_2_/Si substrates, indicating that biaxial strain strongly modifies the bandgap of InSe. Interestingly, we have found a clear thickness dependence on the bandgap strain tunability: thinner flakes are more sensitive to strain than thicker flakes. Figure 3b summarizes the PL shift rate measured for 19 InSe flakes (10 on PC and 9 on SiO_2_/Si) 5 to 30 layers thick. By subtracting the two trends obtained for the PC and the SiO_2_/Si substrates we can obtain the thickness dependent bandgap gauge factor, i.e., the change of bandgap per % of biaxial strain, of InSe that ranges from 195 ± 20 meV %^−1^ (for 5 layers thick InSe) to 63 ± 6 meV %^−1^ (for 30 layers thick InSe). This value is among one of the largest reported values for 2D semiconducting materials so far, as shown in Table 1 and Figure 3d. Interestingly, for ultrathin flakes our bandgap gauge factor is nearly twice that of uniaxial strained InSe,^[^ ^9ab,ac^ ^]^ in very good agreement with recent DFT predictions.^[^ ^11a^ ^]^

**Figure 3:**
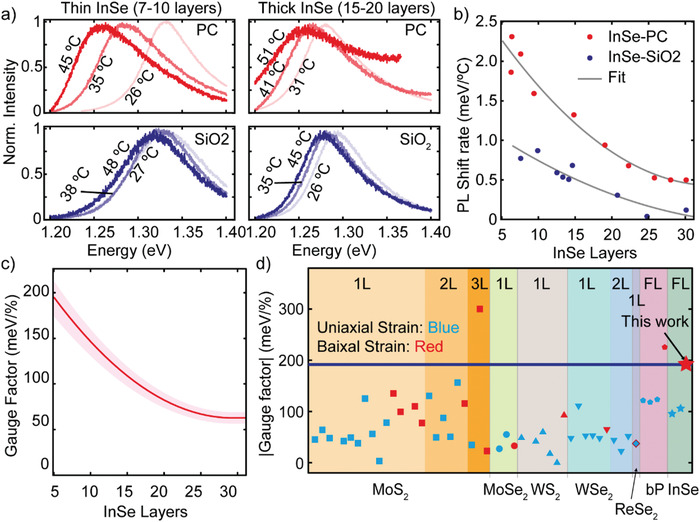
Gauge factor of biaxial strained InSe. a) Photoluminescence spectra of thin (7–10 layers, left) and thick (15–20 layers, right) InSe flakes deposited on PC substrate (top) and on 280 nm SiO_2_/Si substrate (bottom) recorded as a function of temperature (from RT to ≈ 50 °C). b) PL energy shift rate versus thickness of InSe flakes deposited on PC substrate (red) and on 280 nm SiO_2_/Si (blue) substrate. The solid lines represent the best fit for each dataset to a second order polynomial function. c) Calculated gauge factor of biaxial strained InSe flakes as a function of layer numbers. d) The different values of the strain tunability gauge factors of 2D semiconducting flakes reported in the literature with various approaches are compared with the value of biaxial strained InSe obtained in this work (≈200 meV %^−1^).

We further study the effect of biaxial strain on the electronic properties on the Au–InSe–Au photodetector devices in the dark state. The details of basic optoelectronic characterizations of the annealed Au–InSe–Au device on PC and on SiO_2_/Si substrates are shown in Figures S2 and S3 in the Supporting Information, respectively. **Figure** **4**a shows the current versus voltage characteristics (*I*–*V*s hereafter) in linear scale as a function of the PC substrate temperature from ≈23 to ≈ 100 °C, leading to a biaxial strain in the 0–0.48% range.^[^ ^5a^ ^]^ A significant increase in the slope of *I*–*V*s with temperature increase is observed, indicating an increase of conductivity of the device upon substrate thermal expansion (in good agreement with the observed bandgap reduction under biaxial tension). The data is also plotted in semilogarithmic scale (with current absolute value) to facilitate the quantitative comparison between different datasets. The current at *V* = −1 V increases dramatically: from −0.6 pA at ≈23 °C (0% strain) to –0.33 nA at 100 °C (0.48% strain), see inset in Figure 4a. In order to estimate the intrinsic contribution of the temperature increase (without strain) on the observed current change we repeat the measurement on a control device fabricated on a SiO_2_/Si substrate (with very small thermal expansion) finding a negligible current change (Figure 4b and inset). A minor increase of the current value from −0.46 to −2.6 pA at −1 V, due to the increase of thermal excited carriers, is observed.^[^ ^24^ ^]^ We thus attribute the observed current change in the PC device to a piezoresistive response of InSe to biaxial strain. In order to quantify this piezoresistive response, and to compare it with that of other materials, in Figure 4c, we extract the current absolute value flowing through the devices at 1 and −1 V both on PC and SiO_2_/Si substrate as a function of temperature. The calculated electrical gauge factor in our device, GF  =  (*I* − *I*_0_)/*εI*_0_, reaches values of ≈450 at 1 V and ≈1076 at −1 V, ≈5–12 times larger than that found for InSe under uniaxial strain loading and other strained 2D materials. In fact, GF values of ≈150, ≈220 and ≈40 have been reported for single‐, bi‐ and tri‐layer MoS_2_, respectively,^[^ ^6c^ ^]^ upon biaxial strain and GF ≈15–30 for tri‐layer MoS_2_ upon uniaxial strain.^[^ ^25^ ^]^ For graphene sensors GF values up to ≈125 has been reported.^[^ ^6^, ^9^, ^26^ ^]^ Moreover, the large gauge factor and mechanical resilience of 2D InSe makes it even more suitable as a biaxial strain sensor than state‐of‐the‐art silicon strain sensors (GF ≈ 200) with a fracture strain of only ≈0.7%.^[^ ^12^, ^27^ ^]^

**Figure 4:**
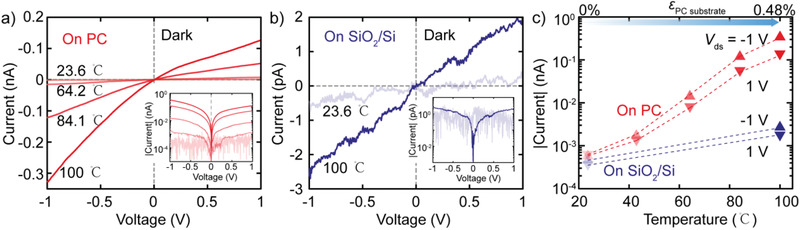
Temperature‐dependent dark current–voltage (*I*–*V*) characteristics of InSe device fabricated on PC and on 280 nm SiO_2_/Si substrate. *I*–*V* curves of Au–InSe–Au device on a) PC substrate and b) on 280 nm SiO_2_/Si substrate recorded in dark conditions as a function of temperature (from ≈23 to ≈ 100 °C) in linear scale and semilogarithmic scale (insets). c) Temperature‐dependency of absolute values of the current flow through Au–InSe–Au devices at 1 and −1 V on PC (red) and on 280 nm SiO_2_/Si (blue) substrate. The top axis in (c) indicates the biaxial strain induced by the thermal expansion of the PC substrate.

The strong PL shift upon biaxial straining indicates that biaxial strain could be an efficient strategy to tune the spectral bandwidth of InSe based photodetectors. In order to study this possibility we measure the photocurrent of the InSe photodetector upon illumination with different wavelengths at a fixed bias of 1 V and power density of 35.4 mW cm^−2^. We address the reader to the Experimental Section for details about the measurement configuration. **Figure** **5**a shows the photocurrent spectra measured at different temperatures between ≈23 and ≈100 °C (corresponding to a biaxial strain range of 0% to 0.48%) in the InSe photodetector fabricated on PC.^[^ ^5^, ^9^ ^]^ The overall spectra redshift upon biaxial strain, as expected from the strain‐induced reduction of the bandgap observed in the PL measurements. This can be seen more clearly in the Tauc plot representation (see inset) that allows for an estimation of the energy cut‐off (Δ*E*_cut‐off_) of the photodetector (the minimum detectable photon energy). The theoretical foundation of this technique (Tauc plot extrapolation) is based on the energy dependence of the above‐bandgap absorption, which appears either as a square relation (direct‐allowed‐transition dominant) or a square‐rooted relation (indirect‐allowed‐transition dominated) and due to the direct bandgap of thin InSe we take a squared plot relation.^[^ ^23^, ^28^ ^]^ In order to determine whether the observed bandgap reduction is mainly caused by the biaxial strain induced by substrate expansion, and not by the temperature increase, we perform again another control measurement on an InSe device fabricated on a SiO_2_/Si substrate. Figure 5b shows how in the control device (with negligible thermal expansion) the redshift of the spectra is less pronounced (another example is shown in Figure S11 in the Supporting Information). Therefore, we can reliably extract the biaxial strain tunability of the energy cut‐off (Δ*E*_cut‐off_) directly from the measurements on the InSe device fabricated on PC. Figure 5c summarizes the energy cut‐off (Δ*E*_cut‐off_) values extracted for the different biaxial strain levels showing a marked linear trend. The slope of this linear relationship is higher than the bandgap gauge factor giving a value of ≈360 meV %^−1^. This could be due to the intrinsic higher uncertainty in the Tauc plot extrapolation method (given the reduced number of data points at the absorption edge part of the spectra).

**Figure 5:**
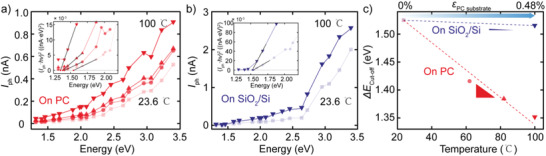
Temperature‐dependence of photocurrent spectra of InSe devices on PC and on 280 nm SiO_2_/Si substrate. a,b) Photocurrent (*I*_ph_) versus illumination photon energy spectra recorded under a fixed illumination power intensity (35.4 mW cm−^2^) at 1 V as a function of temperature (from ≈23 to ≈ 100 °C) of Au–InSe–Au device on a) PC and on b) 280 nm SiO_2_/Si substrate. Insets: Tauc plots: (*I*_ph_ *hν*)^2 ^versus photon energy. c) Temperature‐dependence of bandgap values extracted from the Tauc plots as a function of temperature based on the Au–InSe–Au devices on PC (red) and on 280 nm SiO_2_/Si (blue) substrate. The top axis in (c) indicates the biaxial strain induced by the thermal expansion of the PC substrate.

In summary, we have studied the effect of biaxial strain on the vibrational, photoluminescence, electrical and optoelectronic properties of ultrathin InSe. We found a strong shift of the photoluminescence spectra upon biaxial strain with a gauge factor ranging from 195 meV %^−1^ for five thick layers InSe to 63 meV %^−1^ for 30 layers thick InSe. We also found a giant piezoresponse with an electrical gauge factor of ≈1000 in the dark state. Interestingly, we also demonstrate how the strain tunable bandgap can be exploited to tune the spectral response of InSe photodetectors. This work demonstrates the potential of InSe for future straintronic devices like optical modulators or photodetectors with a strain tunable spectral range.

## Experimental Section

##### Sample Fabrication

Thin InSe and h‐BN flakes were mechanically exfoliated out of high‐quality bulk single crystals. InSe bulk crystals were grown by Bridgman method and h‐BN single crystals were provided by HQ Graphene. During the mechanical exfoliation process, Scotch tape, Nitto tape (Nitto Denko SPV 224) and a Gel‐Film (Gel‐Pak, WF 6.0 mil × 4) stamp were used as reported somewhere else.^[^ ^14a,c^ ^]^ An optical microscope (Motic BA310 Met‐T) in transmission mode was used for flake inspection and to select thin InSe flakes. Selected flakes were deterministically transferred to bridge the 50 nm Au/5 nm Ti electrodes prepatterned on the PC and 280 nm SiO_2_/Si substrates. Then a h‐BN flake was transferred onto the surface to realize the full encapsulated devices. The prepatterned electrodes were fabricated by e‐beam evaporation of 5 nm Ti + 50 nm Au through a metal shadow‐mask (E321 Osilla). Note that all the channel length of Au–InSe–Au devices is ≈30 µm and all the fabrication steps were carried out under ambient conditions.

##### Raman Spectroscopy and PL Measurements

Both the temperature‐dependent Raman and photoluminescence characterization of InSe flakes on PC and on 280 nm SiO_2_/Si substrates were carried out with a confocal Raman microscopy system (MonoVista CRS+ from Spectroscopy & Imaging GmbH). The Raman and PL spectra were recorded using a 532 nm excitation laser at the incident power of 196 µW and a 50× objective with the integration time of 20 s (Raman) and 6 s (PL), respectively.

##### Electronic and Optoelectronic Characterization

Au–InSe–Au devices were characterized in a homebuilt probe station mounted inside a high‐vacuum chamber reported somewhere else.^[^ ^29^ ^]^ The electrical measurements (*I*–*V*, *I–t*) were performed with a source‐measure unit (Keithley 2450). Fiber‐coupled light emitting diodes (LEDD1B‐T‐Cube LED driver, Thorlabs) with wavelength from 365 to 940 nm, were coupled to a multimode optical fiber and projected onto the sample surface by a zoom lens, creating a light spot on the sample with the diameter of 600 µm. Note that all the temperature control from ≈23 to ≈100°C was realized by using a resistance ceramic miniature‐heater (10 mm × 10 mm) mounted on the sample stage connected to current source and a thermocouple to display the temperature.

##### AFM Measurements

The thickness of thin InSe flakes was measured by an ezAFM (by Nanomagnetics) atomic force microscope operated in dynamic mode. The cantilever used was Tap190Al‐G by BudgetSensors with force constant 40 Nm^−1^ and resonance frequency 300 kHz.

## Conflict of Interest

The authors declare no conflict of interest.

## Supporting information

Supporting Information
