# Supplementary material for: Giant Piezoresistive Effect and Strong Bandgap Tunability in Ultrathin InSe upon Biaxial Strain
Source: Adv Sci (Weinh). 2020 Aug 20;7(20):2001645. doi: 10.1002/advs.202001645 (PMC7578899; doi:10.1002/advs.202001645)
Supplement: Supplementary file 1 — Supporting Information [file ADVS-7-2001645-s001.pdf]

© 2020 Wiley-VCH GmbH

ADVANCED SCIENCE
Open Access

Supporting Information

for *Adv. Sci.,* DOI: 10.1002/advs.202001645

Giant Piezoresistive Effect and Strong Band Gap Tunability in Ultrathin InSe upon Biaxial Strain

Qinghua Zhao, Tao Wang*, Riccardo Frisenda*, Andres Castellanos-Gomez*

Supporting Information:

Giant Piezoresistive Effect and Strong Band Gap Tunability in Ultrathin InSe upon Biaxial Strain

Qinghua Zhao, Tao Wang, Riccardo Frisenda*, Andres Castellanos-Gomez**

Q. Zhao, Prof. T. Wang
State Key Laboratory of Solidification Processing, Northwestern Polytechnical University, Xi'an, 710072, P. R. China
Key Laboratory of Radiation Detection Materials and Devices, Ministry of Industry and Information Technology, Xi'an, 710072, P. R. China
E-mail: taowang@nwpu.edu.cn

Q. Zhao, Dr. R. Frisenda, Dr. A. Castellanos-Gomez
Materials Science Factory. Instituto de Ciencia de Materiales de Madrid (ICMM-CSIC), Madrid, E-28049, Spain.

E-mail: riccardo.frisenda@csic.es; andres.castellanos@csic.es

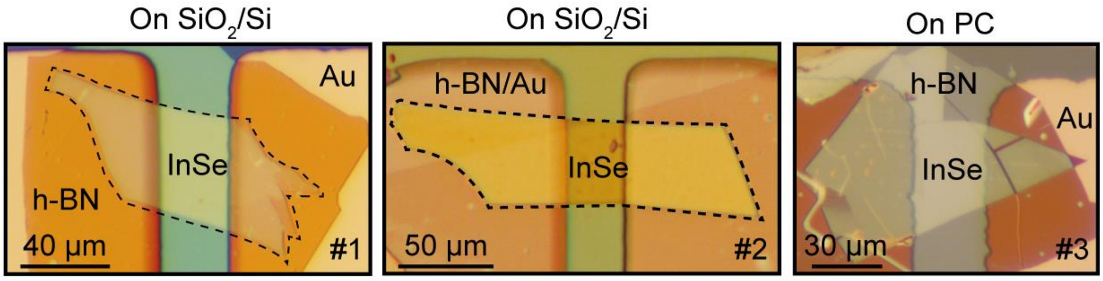

Figure S1. Three Au-InSe-Au devices fabricated on 280 nm SiO_2/Si (#1 and #2) and PC (#3) substrate.

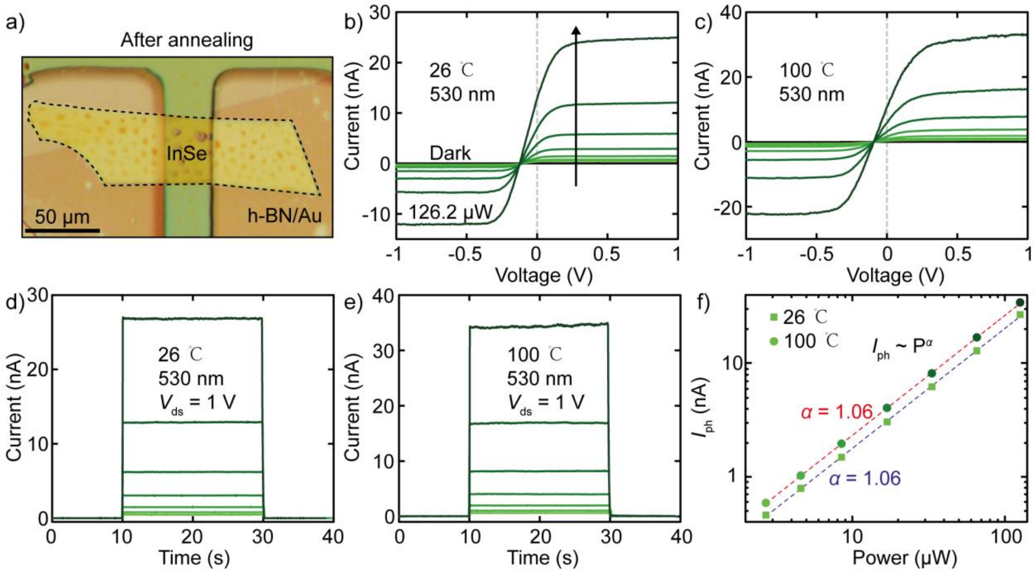

Figure S2. Optoelectronic characterization of device #2 after *in situ* annealing. a) The optical picture of device #2 after annealing. b, c) *I-V* curves recorded in dark condition and as a function of 530 nm illumination power at 26 ℃ and 100 ℃. d, e) *I-t* curves at 1 V recorded as a function of 530 nm illumination power at 26 ℃ and 100 ℃. f) Photocurrent value in the device at 1 V at 26 ℃ (square) and 100 ℃ (circle) *versus* illumination power plot in log-log scale.

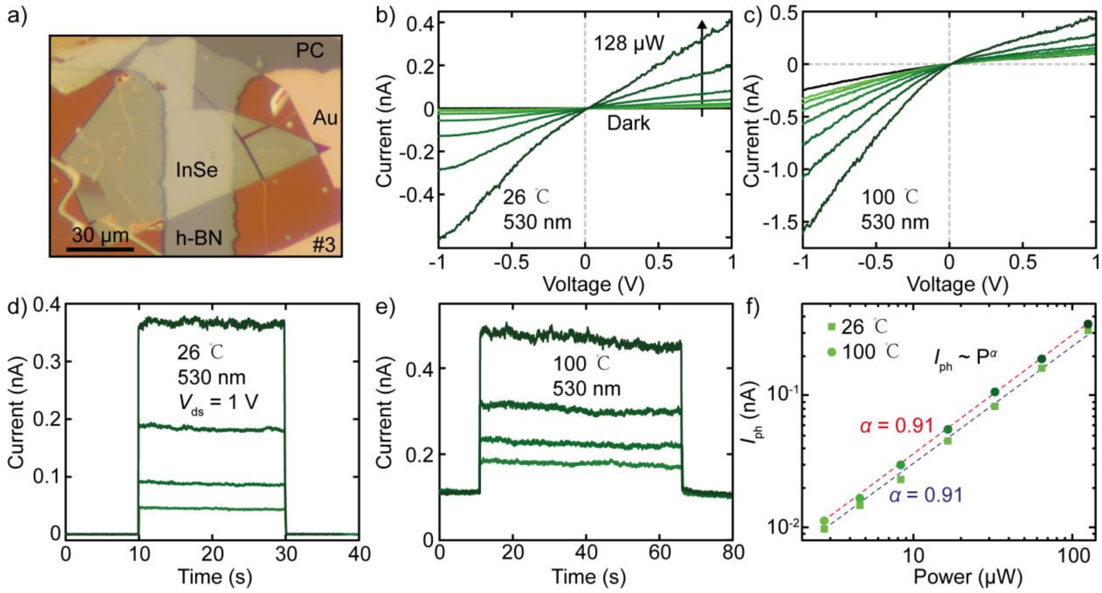

Figure S3. Optoelectronic characterization of device #3 after *in situ* annealing. a) The optical picture of device #3. b, c) *I-V* curves recorded in dark condition and as a function of 530 nm illumination power at 26 ℃ and 100 ℃. d, e) *I-t* curves at 1 V recorded as a function of 530 nm illumination power at 26 ℃ and 100 ℃. f) Photocurrent value in the device at 1 V at 26 ℃ (square) and 100 ℃ (circle) *versus* illumination power plot in log-log scale.

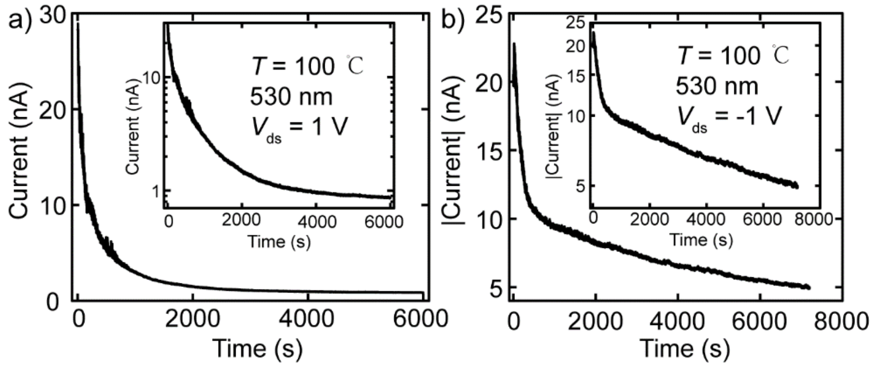

Figure S4. The current evolution during the *in situ* annealing. a, b) The current flowing through the device #1 at 1 V (a) and device #3 at -1 V (b) as a function of *in situ* annealing time in linear and semi-logarithmic (inset) scale.

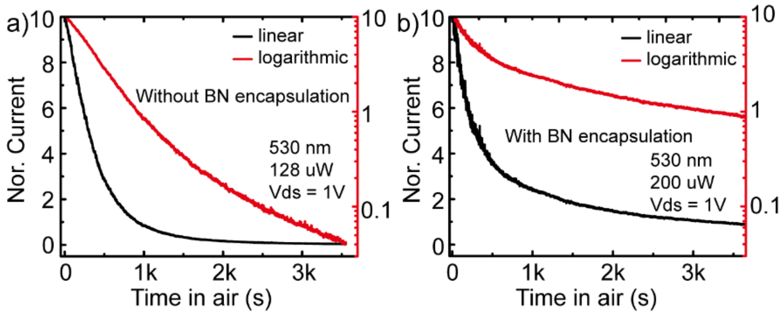

Figure S5. The current evolution during the *in situ* annealing of InSe devices without (a) and with (b) h-BN encapsulation.

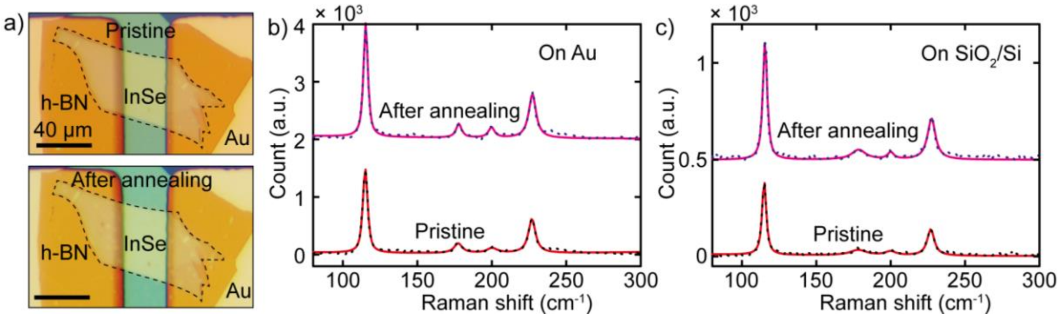

Figure S6. Structural stability before and after *in situ* annealing of Au-InSe-Au device. a) The optical picture of a Au-InSe-Au devices fabricated on SiO_2/Si substrate before and after annealing. b, c) Raman Spectra of InSe flake on Au (b) and on SiO_2/Si (c) recorded in pristine state and after annealing at room temperature.

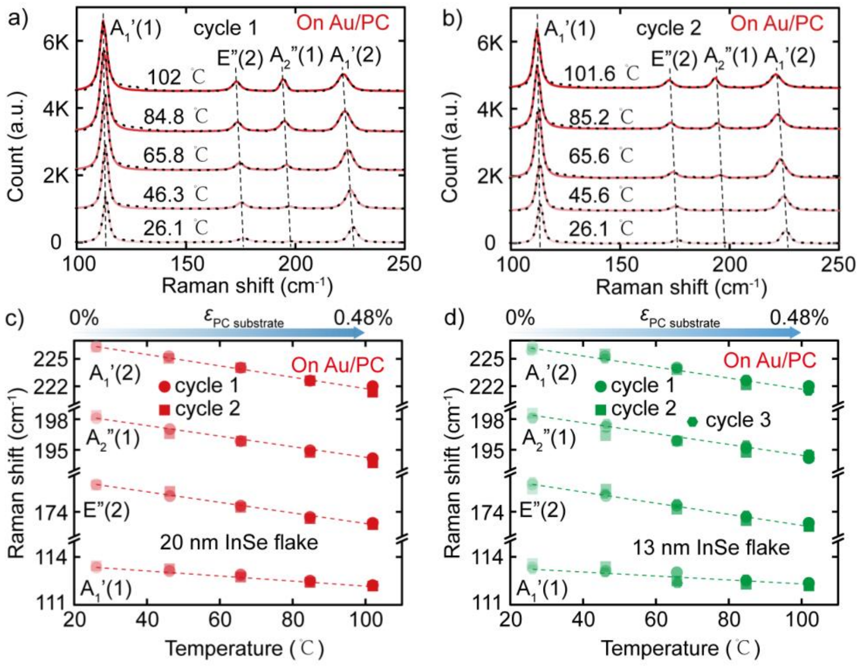

Figure S7. Temperature-dependent Raman Spectroscopy of thin InSe flakes recorded during different heating cycles. a, b) Raman spectra of thin InSe (~20 nm) recorded on Au/PC substrates during heating cycle 1 (a) and cycle 2 (b) with 50× objective as a function of temperature (from ~26℃ to ~ 100℃). c-d) Temperature-dependency of four Raman active modes (A_1’(1), E’’(2), A_2’’(1) and A_1’(1)) of 20 nm InSe (c) and 13 nm InSe (d) on PC substrate during different heating cycles. The top axis indicates the biaxial strain induced by the thermal expansion of the PC substrate.

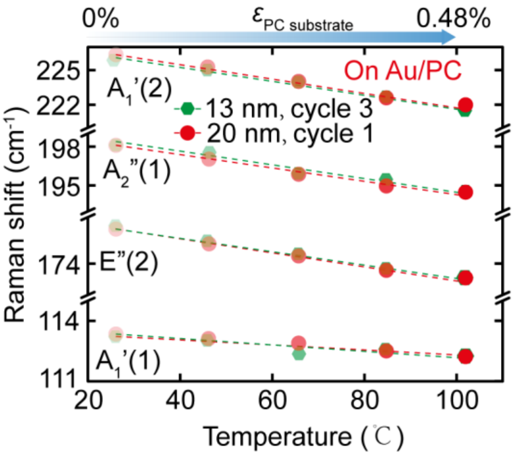

Figure S8. Temperature-dependency of four Raman active modes (A_1’(1), E’’(2), A_2’’(1) and A_1’(1)) of two thin InSe flakes with the thicknesses of 13 nm (green) and 20 nm (red) on PC substrate. The top axis in (c) indicates the biaxial strain induced by the thermal expansion of the PC substrate.

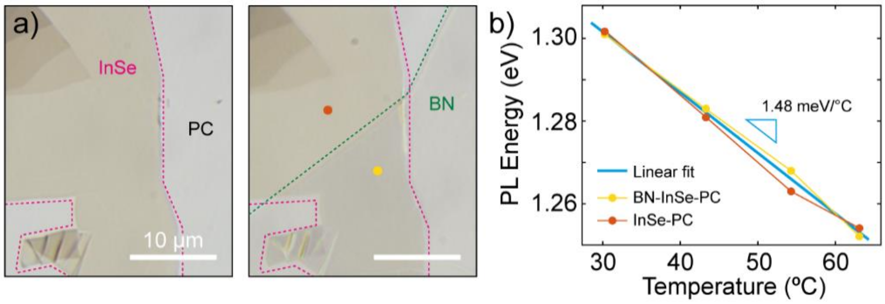

Figure S9. The effects of h-BN encapsulation on band gap tunability of biaxial strained InSe. (a) A thin InSe flake (~13 layers) deposited on PC substrate before (left) and after (right) being half encapsulated with h-BN. (b) The PL energy as a function of temperature of the InSe flake recorded at the two positions indicated by the colored circles in panel (a) corresponding to InSe-PC and BN-InSe-PC. The blue line is a linear fit to the data with slope 1.48 meV/°C.

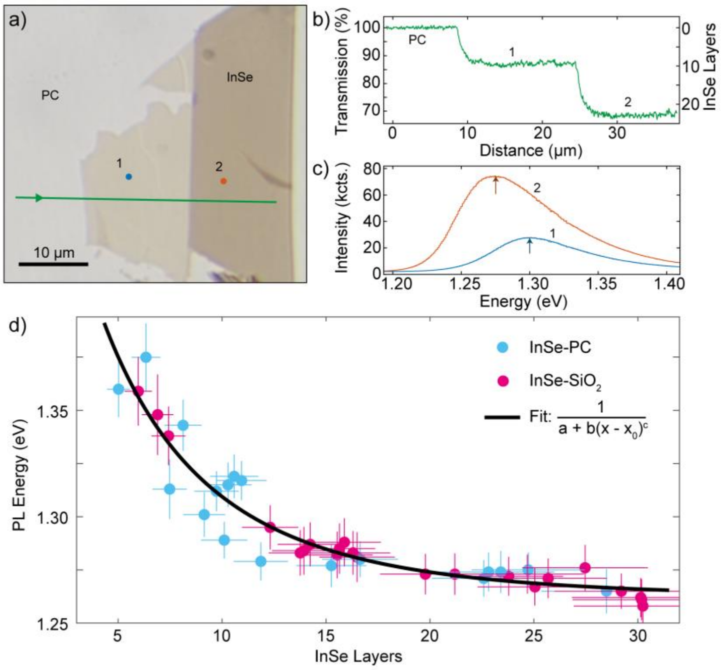

Figure S10. The PL energy versus InSe flake thickness. (a) Optical picture of a thin InSe flake with two different regions (marked by 1 and 2) deposited on PC substrate and recorded under transmission mode. b) Total transmission of the blue channel of the picture in panel (a) taken along the green line, with the PC having unitary transmission. The right axis indicates the corresponding estimated number of layers of the InSe flake. c) Photoluminescence spectra of regions 1 and 2 recorded at the locations marked by the red and blue circles in panel (a). d) Center of the PL peak as a function of InSe flake thickness extracted from 42 different InSe flakes deposited on PC and SiO_2. The black line is the best fit to the function shown in the legend with parameters: a=0.79, b=-$1.3 \cdot 10^7$ c=-5.5, x_0=-26.

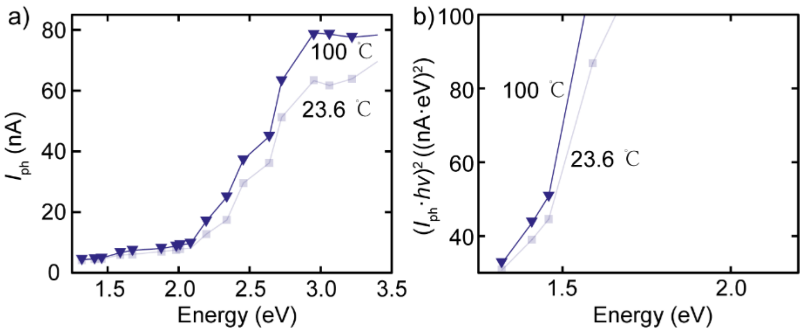

Figure S11. Temperature-dependency of photocurrent spectra (a) and Tauc plot (b) of device #2.
